# Strong piezoelectricity in single-layer graphene deposited on SiO_2_ grating substrates

**DOI:** 10.1038/ncomms8572

**Published:** 2015-06-25

**Authors:** Gonçalo da Cunha Rodrigues, Pavel Zelenovskiy, Konstantin Romanyuk, Sergey Luchkin, Yakov Kopelevich, Andrei Kholkin

**Affiliations:** 1 Department of Materials and Ceramic Engineering, CICECO, University of Aveiro, Aveiro 3810-193, Portugal; 2 Institute of Natural Sciences, Ural Federal University, Ekaterinburg 620000, Russia; 3 Instituto de Fisica, UNICAMP, Campinas, São Paulo 13083-859, Brazil

## Abstract

Electromechanical response of materials is a key property for various applications ranging from actuators to sophisticated nanoelectromechanical systems. Here electromechanical properties of the single-layer graphene transferred onto SiO_2_ calibration grating substrates is studied via piezoresponse force microscopy and confocal Raman spectroscopy. The correlation of mechanical strains in graphene layer with the substrate morphology is established via Raman mapping. Apparent vertical piezoresponse from the single-layer graphene supported by underlying SiO_2_ structure is observed by piezoresponse force microscopy. The calculated vertical piezocoefficient is about 1.4 nm V^−1^, that is, much higher than that of the conventional piezoelectric materials such as lead zirconate titanate and comparable to that of relaxor single crystals. The observed piezoresponse and achieved strain in graphene are associated with the chemical interaction of graphene's carbon atoms with the oxygen from underlying SiO_2_. The results provide a basis for future applications of graphene layers for sensing, actuating and energy harvesting.

## Abstract (web summary)

Pristine graphene is not piezoelectric, but adsorbing dopant atoms can induce a piezoresponse by breaking the material's inversion symmetry. Here the authors demonstrate strong out-of-plane piezoelectricity in graphene on a silica substrate, providing a system for two-dimensional sensing and actuation.

## Graphical abstract

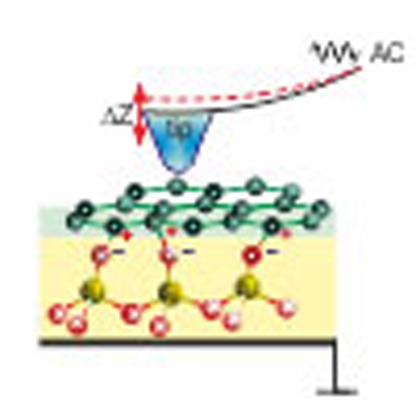

Recent discovery of piezoelectricity in two-dimensional (2D) materials^1^,^2^ opens up new opportunities for stretchable electronics^3^, sensors, actuators and other electronic components based on the direct and converse piezoelectric effects. Being 2D monoatomic material with many unique properties^4^, graphene is one of the favourable candidates for these applications. It exhibits a variety of emergent properties such as high thermal conductivity^5^,^6^, superior mechanical strength and extremely high flexibility^7^,^8^. Although pristine graphene does not possess any piezoelectric activity due to its intrinsically centrosymmetric crystal structure, piezoelectricity can be induced by breaking of the inversion symmetry by adsorption of foreign atoms, by introducing specific in-plane defects or by non-uniform deformation of graphene layers in which strain gradients create internal polarization in a material^2^,^9^,^10^,^11^,^12^. The predicted piezoelectricity of modified graphene is high enough and comparable to that in conventional piezoelectric materials^9^. However, only theoretical predictions have been published so far, which provided the expected values of piezoelectric coefficients. One of the requirements for any form of graphene to exhibit piezoelectricity is its semi-conducting or insulating state. It has been shown that the engineered strain in graphene leads to opening/tuning of the band gap and, therefore, graphene can evolve from semi-metal to semi-conducting state^12^,^13^. In theory, if graphene (centrosymmetric) is placed in a symmetrical strain field, net polarization will remain zero, and the apparent piezoelectric response will vanish. Therefore, a non-symmetric strain field (or strain gradient) is needed for the non-zero net polarization and for the consequent apparent piezoelectricity of graphene^2^.

Lee *et al.*^14^ demonstrated that the anisotropic strain geometry of a graphene layer deposited on top of an underlying grid structure is defined by the period of the substrate. This was confirmed by the polarization-dependent Raman spectra^14^. Another effect in periodically modulated graphene on the SiO_2_ grating (predicted theoretically) was periodically localized doping induced by chemical interaction of graphene with SiO_2_ surface^14^,^15^,^16^,^17^. This interaction with underlying substrate atoms can also induce the band gap opening^18^, the net dipole moment and polarization in the graphene layer.

In this work, we present the results of experimental study of single-layer graphene (SLG) deposited on SiO_2_ calibration grating substrates by piezoresponse force microscopy (PFM) and confocal Raman spectroscopy. We experimentally confirm the existence of electric polarization induced in graphene deposited on SiO_2_ via direct measurements of converse piezoelectric effect by PFM. Piezoelectric activity was mainly observed on the supported graphene regions where van der Waals and/or chemical interaction between the SiO_2_ surface and graphene layer can induce an anisotropic strain and detectable PFM signal. The in-plane strain in graphene was evaluated by polarization-dependent Raman spectroscopy.

## Results

### Materials and experimental set-up

SLG was grown by chemical vapour deposition on a copper substrate and then transferred by the wet process on top of the Si/SiO_2_ calibration grating TGZ4 by the company 2D-Tech (UK) The substrate was a commercially available standard calibration grating for atomic force microscopy (AFM) purchased from NT-MDT (Russia). It consisted of a grid of parallel rectangular pitches with the height 1,317±10 nm and period 1,500±10 nm. The thickness of the thermally grown SiO_2_ layer was 1,400±10 nm.

Topography and PFM measurements were performed using a Ntegra Prima AFM (NT-MDT, Russia) equipped with the lock-in amplifier (SR-830, Stanford Research, USA) and function generator (FG-110, Yokohama, Japan). The measurements were carried out using Pt-coated cantilevers purchased from NT-MDT (Russia): NSG30/Pt (resonance frequency 270 kHz, force constant 40 N m^−1^) and CSG30/Pt (resonance frequency 42 kHz, force constant 0.6 N m^−1^). Standard topography mapping was carried out in a tapping mode. PFM and conductive AFM were performed using CSG30/Pt cantilevers in the contact mode. PFM measurements were carried out in a quasi-static regime at the frequencies <100 KHz with the 0–5 V amplitude of a.c. excitation signal under a vacuum ∼10^−1^–10^−2^ torr. Frequency-dependent measurements of PFM amplitude were carried out in the 10–900 kHz frequency range. Raman measurements were performed using a confocal Raman microscope WiTec alpha300AR (WiTec GmbH, Germany). A 488-nm solid-state excitation laser with polarization along the grating stripes was focused by a 100 × objective (numerical aperture=0.75). Spectral resolution of the used diffraction grating was 3.19 cm^−1^. Raman spectra were acquired by a charge-coupled device camera with 0.2 s integration time.

### Measurements

**Figure 1:**
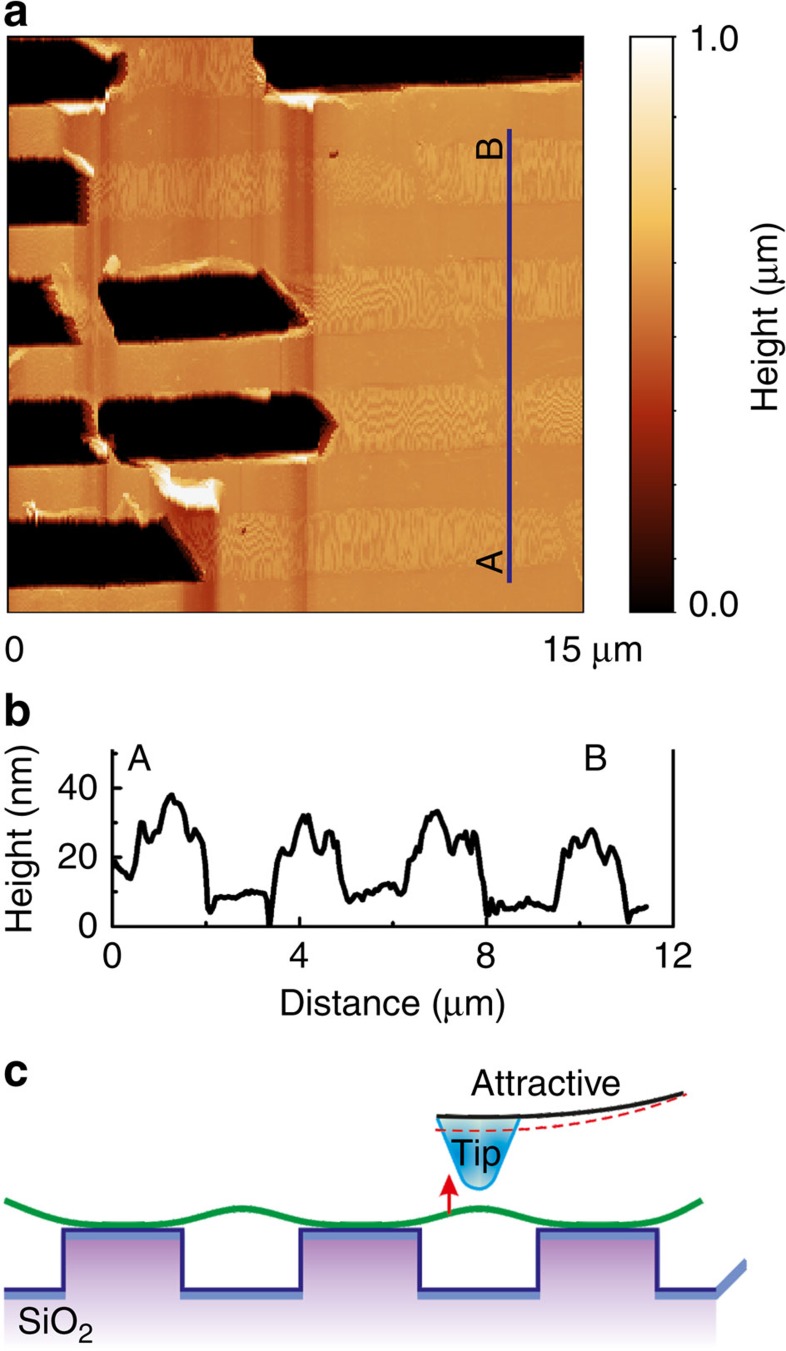
Topography of the sample. (**a**) Measured in the tapping mode topography of SLG on the grating. Left part represents graphene damaged under scanning. (**b**) Cross-section along the line A–B showing supported and suspended graphene areas. Suspended graphene is about 20 nm higher than the supported one. (**c**) Schematic of the graphene and substrate arrangement explaining the origin of the observed bending.

After the deposition of graphene, the topography measurements of the entire structure (supported+suspended graphene) were performed using a conventional AFM tapping mode (Fig. 1). In this mode, the topography of graphene layers remained stable after several consecutive scans, although some of the suspended areas were damaged.

By contrast, scanning in a contact mode required by PFM a deleterious effect on the structure of both supported and suspended areas could be seen. Graphene layer could be destroyed after a single scan even at zero deflection level (minimal force). Such behaviour demonstrated sufficiently low stability of graphene under an influence of lateral (in-plane) force and special care (minimal lateral and vertical forces, orientation of cantilever normal to the grating pitches) was taken to prevent early degradation of the SLG during measurements.

Representative topography of the Si/SiO_2_ calibration grating substrate with the SLG on top is shown in Fig. 1a. In this figure, we can clearly distinguish a height contrast between supported and suspended graphene areas. Higher topography of the suspended graphene as compared with the supported one (cross-section shown in Fig. 1b) can be explained by the apparent bending of SLG under an attractive force exerted by the tip during scanning in tapping mode (schematic arrangement is shown in Fig. 1c).

The mechanical strain state of graphene layer was studied by Raman spectroscopy. Analysis of G-band frequency shift allowed estimating the value and spatial distribution of mechanical strains, as well as strain gradients (Supplementary Figs 1–4).

**Figure 2:**
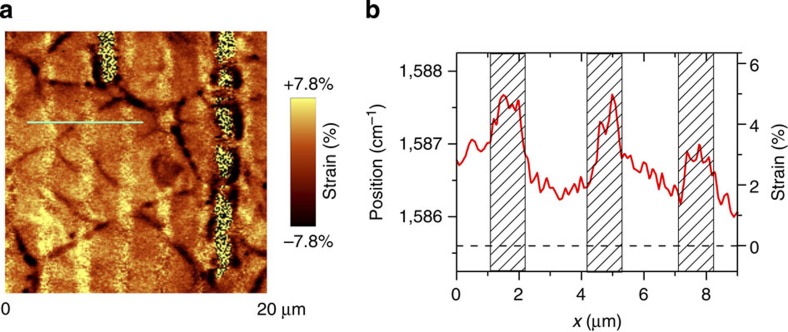
Spatial distribution of strain over the sample structure. (**a**) Strain map of SLG on the Si/SiO_2_ grating. (**b**) Variation of G-band position and strain across the grating (blue line in **a**); shaded rectangles correspond to supported graphene; dashed line denotes the initial (unstrained) value of G-band position.

Spatial distribution of the strains (strain map) is shown in Fig. 2a. Strain over the big area with graphene defects varies in a wide range from −7.8 to +7.8%. Positive sign here corresponds to the tension of the graphene sheet, whereas the negative one shows the compression. The compressive stresses are mainly localized around graphene holes and along the lines imputed to wrinkles.

Tensile strains occur in defect-free regions of the graphene. The largest values of such strains fall on supported graphene (shaded rectangles in Fig. 2b) and are about 2.5 times greater than for suspended regions (about 2%). Strains at the grating ridges are distributed more or less uniformly and demonstrate a sharp decrease at the grating depressions. Using the Raman results, we could evaluate the quality of graphene sheet (Supplementary Note 1). It is demonstrated that the transferred graphene is mainly a single layer.

**Figure 3:**
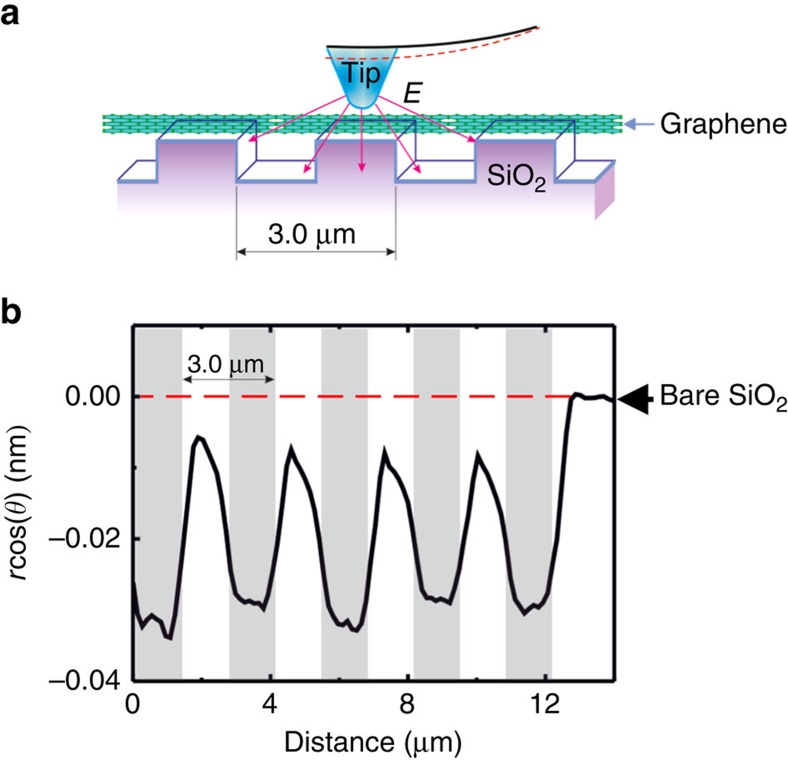
Spatial variation of piezoelectric response. (**a**) Schematic of the PFM measurements on single-layer graphene adsorbed on the TGZ4 grating substrate. (**b**) Cross-section of the piezoresponse along the line on graphene across the grating structure (shaded areas correspond to supported graphene). The dashed red line denotes the baseline corresponding to the signal on the bare SiO_2_ substrate, which is shown as the tail at the right part of **b**. The measurements were carried out at 90 kHz (*V*_a.c._=5 V). The PFM signal phase *θ* is 180° on graphene.

PFM measurements were carried out in a contact mode. First, we applied a conventional low-frequency PFM using high enough a.c. voltage (5 V) applied between the tip and the substrate to detect tiny (pm level) piezoelectric vibrations of the graphene surface. Despite the partial damage, we could find areas where the topography did not change during scanning. The cross-section of the PFM (mixed) signal across such an area is shown in Fig. 3.

Zero PFM signal on the right part of Fig. 3b corresponds to bare SiO_2_ substrate and, thus, provides a natural reference to the PFM signal observed on graphene.

It is seen that clear negative piezoresponse is observed on both supported (shaded areas) and suspended graphene, and the signal on the supported graphene is four times higher than on the suspended one. The amplitude of the electric field-induced vibrations (first harmonic) was about 0.02–0.03 nm. The maximum strain attained and corresponding piezoelectric coefficient will be estimated below.

**Figure 4:**
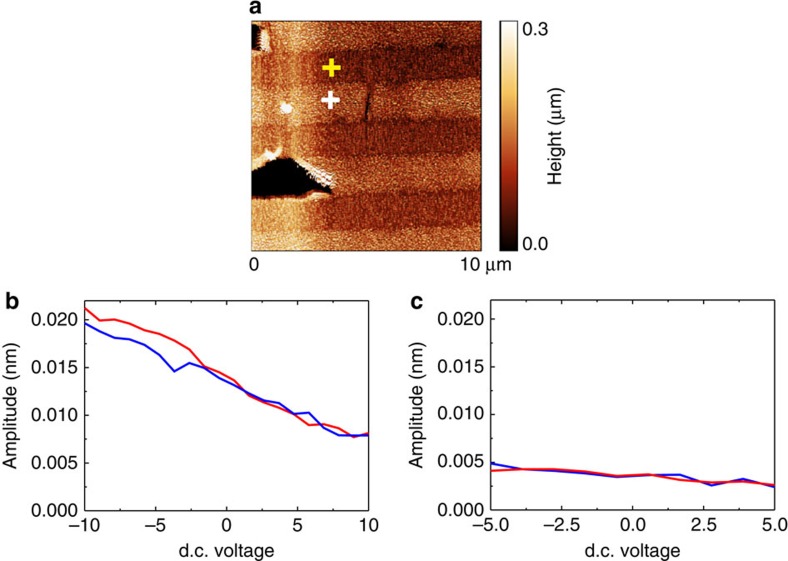
Voltage spectroscopy piezoresponse on supported and suspended graphene. (**a**) Topography of the sample with the tip positions on supported and on suspended graphene marked by yellow and white cross, respectively. (**b**,**c**) Piezoresponse voltage spectroscopy measured in the step mode on supported and suspended graphene, respectively. Driving a.c. voltage is 1 V and measurement frequency is 20 kHz.

To avoid further degradation of the SLG, voltage spectroscopy measurements^18^,^19^,^20^ were performed when the tip was fixed on graphene surface. Voltage spectroscopy measurements were carried out in a piezo-step mode to decrease the measurement time. Measurements performed with the retracted tip showed a typical electrostatic V-shaped response^19^,^20^,^21^. Sweeping d.c. bias in the range −10 V<V<+10 V did not result in any hysteresis and polarization switching as common for ferroelectric materials. On the contrary, typical pyroelectric-type behaviour was observed (Fig. 4) where d.c. bias could only be a source of additional (electric field-induced) polarization. The amplitude of the piezoresponse on the SLG was increased when the negative d.c. bias was applied to the tip and decreased for the opposite direction. The reason for such behaviour will be explained below.

**Figure 5:**
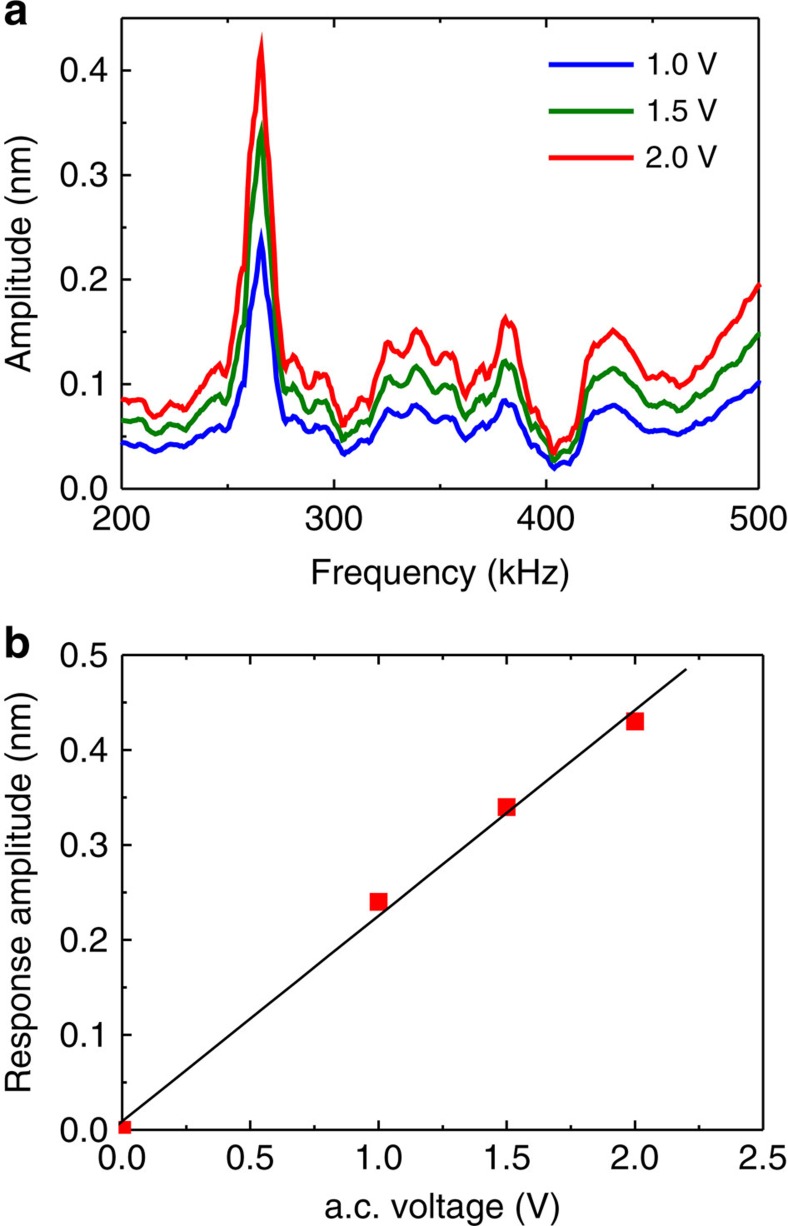
Piezoresponse measured versus a.c. frequency and voltage. (**a**) Frequency dependence of PFM amplitude at different a.c. field levels near the first contact resonance measured on supported graphene layer. (**b**) Piezoresponse amplitude at the resonance as a function of applied a.c. voltage.

To further prove the piezoelectric nature of the response, we measured the frequency dependence of the signal under different a.c. voltage levels. Resonance amplification at the cantilever fundamental resonance was used to get a.c. voltage dependence at low biases and to verify the linearity of the response. Conventional resonance spectra were observed with the fundamental resonance at 262 kHz (Fig. 5a). Signal was amplified at the resonance and demonstrated almost linear behaviour as a function of a.c. amplitude as expected for true piezoelectricity (Fig. 5b). Since no V-shaped response in the voltage spectroscopy measurements was observed (Fig. 4), the electrostatic response was excluded. No sign of non-linearity was observed up to 2 V of a.c. bias, thus, attesting graphene to be a linear pyroelectric and piezoelectric. The resonance frequency of the cantilever on supported graphene was about six times higher than that of the free cantilever resonance (42 kHz). It corresponds to the fundamental resonance for the clamped cantilever^22^.

## Discussion

**Figure 6:**
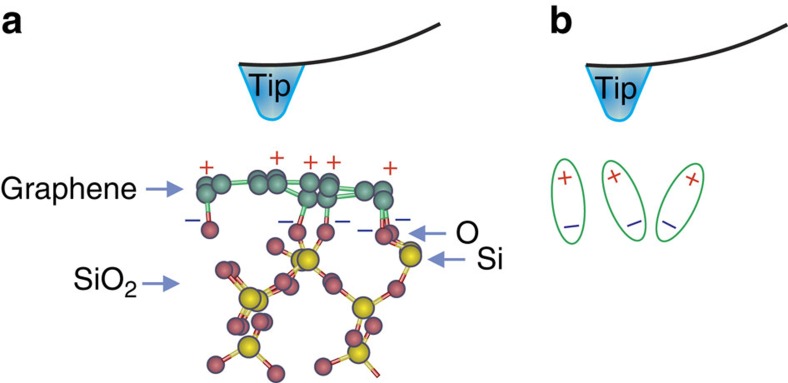
Origin of the measured piezoresponse. (**a**) Schematic of graphene layer on SiO_2_ substrate with oxygen termination. (**b**) Formation of Chemical interaction of carbon and oxygen atoms induces dipolar surface states oriented close to normal of the substrate surface.

The supported and suspended graphene layers possess different chemical structures. We assume that the suspended graphene is a pristine single layer while the supported one can form chemical bonds with the underlying SiO_2_ substrate^15^. The chemical interaction of carbon atoms with oxygen atoms of the substrate can induce non-zero net dipole moment and polarization in the studied system. The mechanism of the polarization is schematically shown in Fig. 6. Due to random orientation of the SiO_2_ complexes on the surface layer, the C^+^–O^−^ dipoles partly deviate from the substrate (Fig. 6b). In this case, we can assume that the application of the negative electric bias to the tip will orient the dipoles closer to the normal direction and increase the net polarization. Application of the positive electric field to the tip will deviate dipoles from the normal direction and decrease the net polarization. As a result, increasing/decreasing of the net polarization results in increasing/decreasing amplitude of the PFM signal as shown in Fig. 4b. Note that the external electric field cannot reorient the fixed dipoles, therefore, no ferroelectric hysteresis could be observed.

We note that the piezoresponse signal distribution shown in Fig. 3b correlates well with the mechanical tensile strain distribution of graphene (Fig. 2b), however, no amplification of the piezoresponse is observed near the grating edges where the maximum strain gradient is expected.

It means that the out-of-plane polarization observed in this work is not coupled with the in-plane symmetry breaking induced by lateral strain in graphene.

The specificity of the considered system where graphene layer can act as a top electrode can explain similar behaviour of PFM response for suspended and supported graphene layers. Specifically, in the supported graphene (with lower conductivity due to oxidation), we can excite and detect surface deflection directly under the tip. When the tip is in contact with the suspended area (with higher conductivity), the electric field is applied over the area covered by the graphene sheet, reaches the dipoles in the supported regions and excites mechanical vibrations. These mechanical vibrations from the supported regions are transferred by the graphene membrane and drive the tip.

Significant frequency shift of the G-band seen in the Raman spectra on the supported regions (Fig. 2b) associated with the high in-plane strain 4.0–5.0% is a result of the described interaction of carbon atoms of graphene with oxygen atoms of SiO_2_. It is known that the strain in graphene or/and additional oxygen-containing functional groups can transfer the graphene into semi-conducting or insulating state^12^,^13^, as in the case of graphene oxide (GO). Simultaneously, the interaction of graphene with SiO_2_ surface can also induce the gap opening^23^. We believe that these effects are responsible for the appearance of polarization and associated piezoelectric effect in supported graphene. However, the origin of the observed strains and piezoelectric activity is chemical (or van der Waals) interaction between the graphene layer and SiO_2_.

Strain induced by the electric field *E* perpendicular to the surface plane can be described as follows:


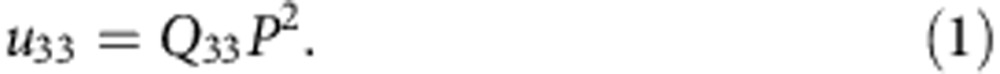


Here the electric polarization *P* is the sum of the induced, *P*_i_, and spontaneous, *P*_s_, polarization: *P*=*P*_i_+*P*_s_=(*ɛ*−1)*ɛ*_0_*E*+*P*_s_. Then equation (1) can be rewritten as:


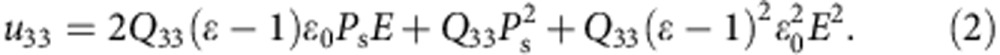


The first term 2*Q*_33_(*ɛ*−1)*ɛ*_0_*P*_s_*E* corresponds to the piezoelectric effect, with the piezoelectric coefficient *d*_33_=2*Q*_33_(*ɛ*−1)*ɛ*_0_*P*_*s*_. The last term, 
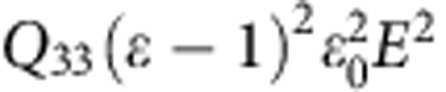
, is an electrostrictive contribution. Leaving only dynamic components depending on the electric field *E* (first and last terms):


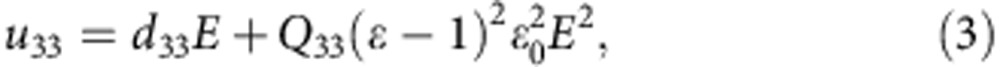


The applied electric field *E* is a combination of a.c. *E*_a.c._ and d.c. *E*_d.c._ components: *E*=*E*_d.c._+*E*_a.c._. Since the applied *E*_a.c._=*E*_0_ cos (*ωt*+*φ*_0_), then normal to the surface displacement, 
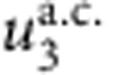
, detected by PFM can be written as: 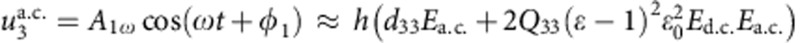, where *h* is thickness of the carbon-oxide dipole layer. The amplitude of the first harmonic thus takes the form:


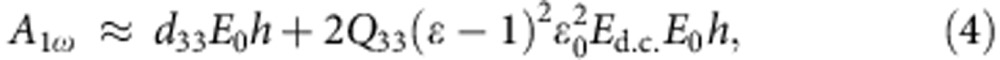


where *E*_0_ is the magnitude of a.c. electric component, *E*_a.c._. For thin carbon-oxide dipole layer with *h*<<*R*_0_, where *R*_0_ is the tip radius, we can consider electric field below the tip in the layer as uniform. Using the relation for tip potential (Supplementary Note 2; Supplementary Equations 5–7) and taking into account that *h*<<*R*_0_ and 
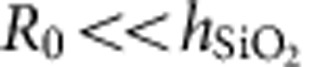
 (Supplementary Figure 5), we can estimate the electric field in carbon-oxide dipole layer as 
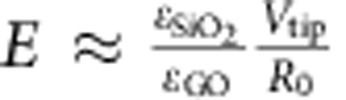
. Then, based on experimental data (Figs 4 and 5), we can estimate the piezoelectric coefficient *d*_33_ as follows: 
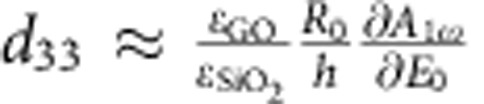
. At low frequency (that is, in the quasi-static regime), we estimated 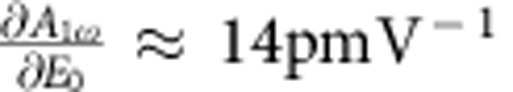. Typical carbon oxygen bond length in the carbonate ions^24^ is in the range 1.2–1.5 Å. Typical distance between graphene layers in graphite is 2.5–3 Å. Assuming *h*=3 Å and *R*_0_=30 nm, we can estimate the longitudinal piezoelectric coefficient of graphene layer 
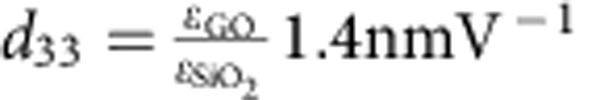
, and 
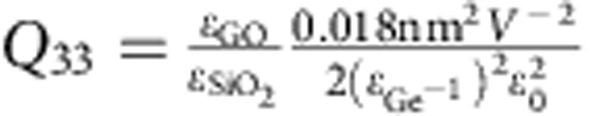
.

Dielectric constant for SiO_2_ is 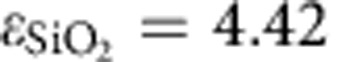 (ref. 25) and for GO is *ɛ*_GO_≈4.3 (ref. 26), then we arrive at the estimation *d*_33_≈1.44 nm V^−1^ and *Q*_33_≈11.4 m^4^ C^−2^. The value of piezoelectric coefficient is more than twice of the best piezoelectric ceramics of the lead zirconate titanate family^27^ and is comparable with the best piezoelectrics of relaxor single crystal family^28^. The value of electrostriction coefficient agrees well with the general trend, that is, increases with the decrease of dielectric constant and increase of elastic compliance^29^. Its value is similar to those of polymers (for example, polyvinyl chloride) and is much smaller than that for ferroelectric perovskites^29^.

When this manuscript was prepared for the submission, the paper by Wang *et al.*^30^ was published and reported an in-plane (band) piezoelectric effect on suspended graphene membranes, which deformation was controlled by the AFM tip pressure and both drain and gate voltages. They, thus, measured the in-plane direct piezoelectric effect in pristine graphene of about 37 nC N^−1^. This value is about 25 times greater than that obtained in our experiments on supported SLG. However, the piezoresponse was measured under non-equilibrium conditions where the converse effect should be small due to high in-plane conductivity. In contrast, we observed a stable static (equilibrium) out-of-plane converse piezoelectric effect on supported graphene induced by underlying SiO_2_. This effect is directly related to a huge number of possible applications such as motors, actuators, resonators and micro- and nanomechanical systems based on graphene. Naturally, the displacement level can be increased by fabricating thin SiO_2_ membrane or bridge structures. As such, a high value of the out-of-plane piezoelectric coefficient observed in our work provides a basis for such applications.

In summary, we observed a strong piezoelectric activity of the SLG deposited on Si/SiO_2_ calibration grating substrates. Mapping the strain distribution in graphene was performed via confocal Raman measurements and converse piezoelectric effect was measured locally by PFM. The piezoelectric activity in graphene layers was attributed to the chemical interaction of graphene atoms with underlying oxygen from SiO_2_ substrate. Piezoelectric effect is sufficiently high (*d*_33_≈1.4 nm V^−1^, that is, more than twice of the best piezoelectric ceramics such as modified lead zirconate titanate). We foresee a number of emergent applications when using graphene/SiO_2_ structures as a platform for future sensors and actuators.

## Additional information

**How to cite this article:** da Cunha Rodrigues, G. *et al.* Strong piezoelectricity in single-layer graphene deposited on SiO_2_ grating substrates. *Nat. Commun.* 7:7572 doi: 10.1038/ncomms8572 (2015).

## Supplementary Material

Supplementary Information Supplementary Figures 1-5, Supplementary Notes 1-2 and Supplementary References
