# Supplementary material for: Strong piezoelectricity in single-layer graphene deposited on SiO2 grating substrates
Source: Nat Commun. 2015 Jun 25;6:7572. doi: 10.1038/ncomms8572 (PMC4491826; doi:10.1038/ncomms8572)
Supplement: Supplementary Information — Supplementary Figures 1-5, Supplementary Notes 1-2 and Supplementary References [file ncomms8572-s1.pdf]

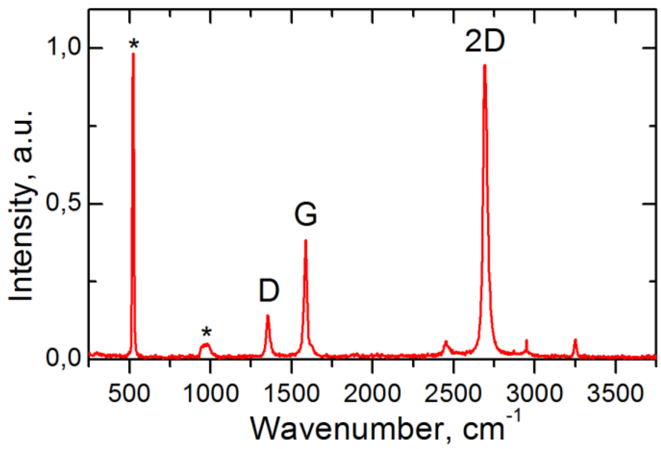

Supplementary Figure 1. Typical Raman spectrum of SLG on the SiO_2 substrate. The D, G and 2D bands are marked. Asterisks denote lines corresponded to the first and the second order Raman scattering of the substrate.

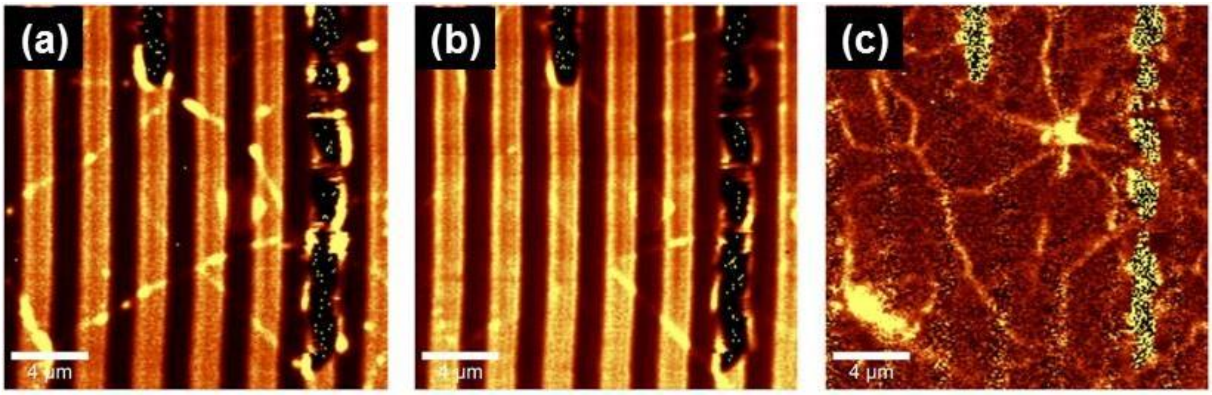

Supplementary Figure 2. Spatial distribution (Raman maps) of integrated intensity of (a) the G-band and (b) the 2D-band. (c) Raman map of ratio of integrated intensities of the D and G-bands.

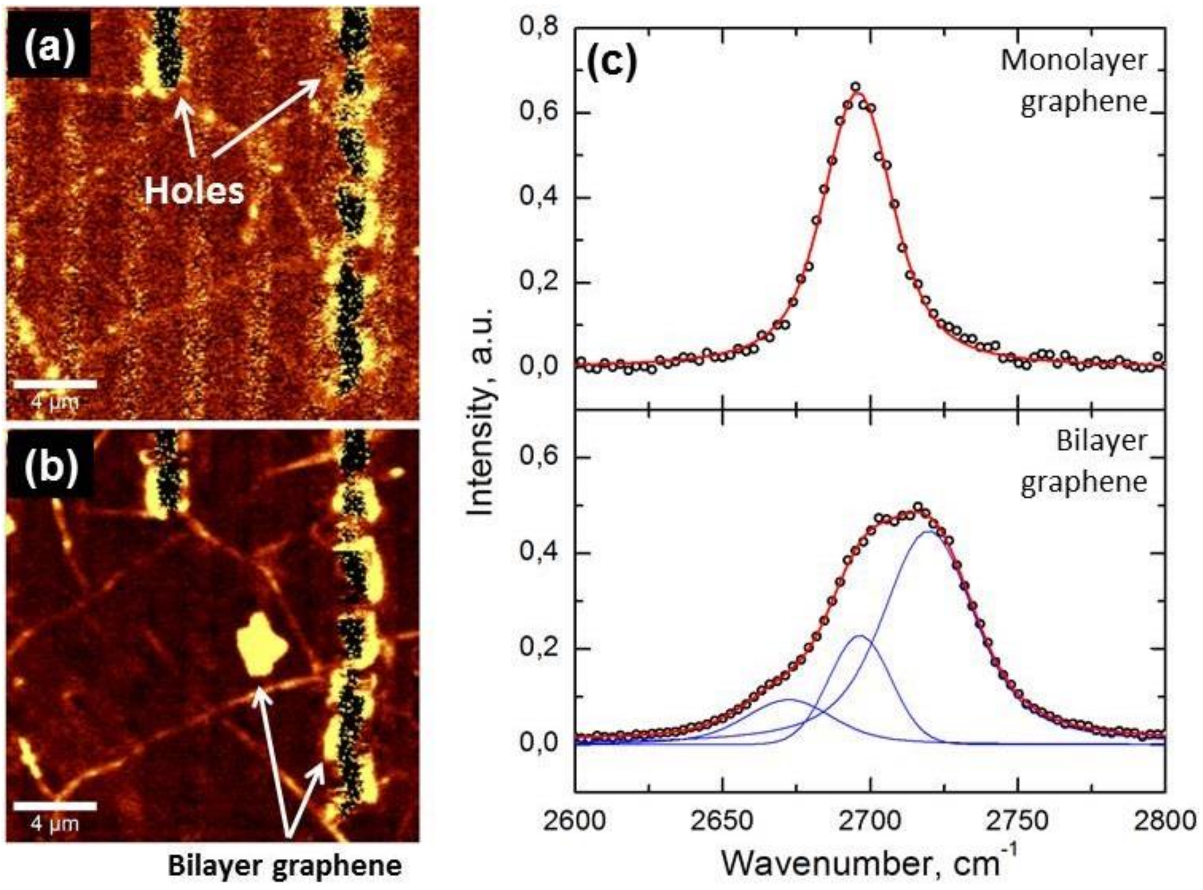

Supplementary Figure 3. Raman maps of FWHM of (a) the G-band and (b) the 2D-band. (c) Shape of the 2D-band outside (top) and inside (bottom) the bright spot in FWHM(2D) map. Open circles correspond to the experimental data; blue lines are the Lorentz fitting (bottom).

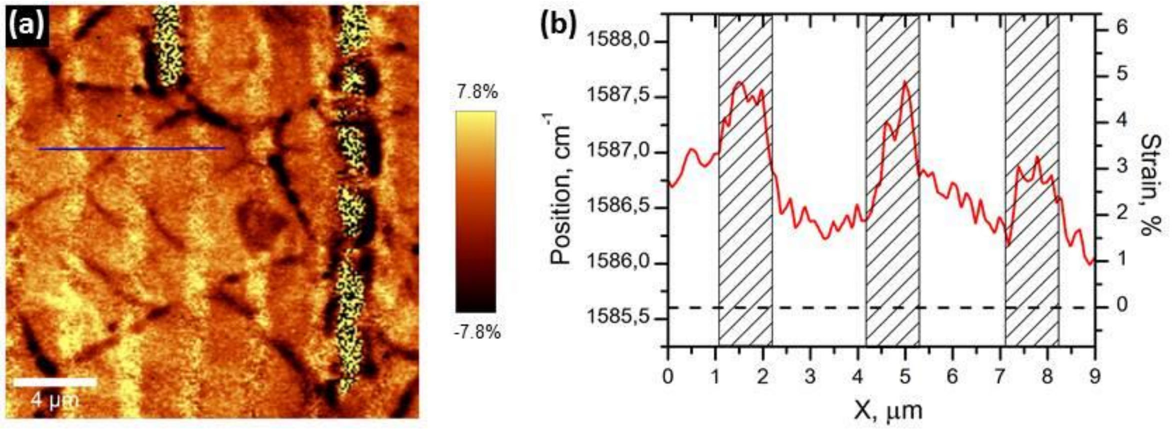

Supplementary Figure 4. Spatial distribution of strain over the sample structure. (a) Strain map of SLG on the silicon grating. (b) Variation of the G-band position and strain across the grating (blue line); shaded rectangles correspond to supported graphene, dashed line denotes the initial (unstrained) value of the G-band position.

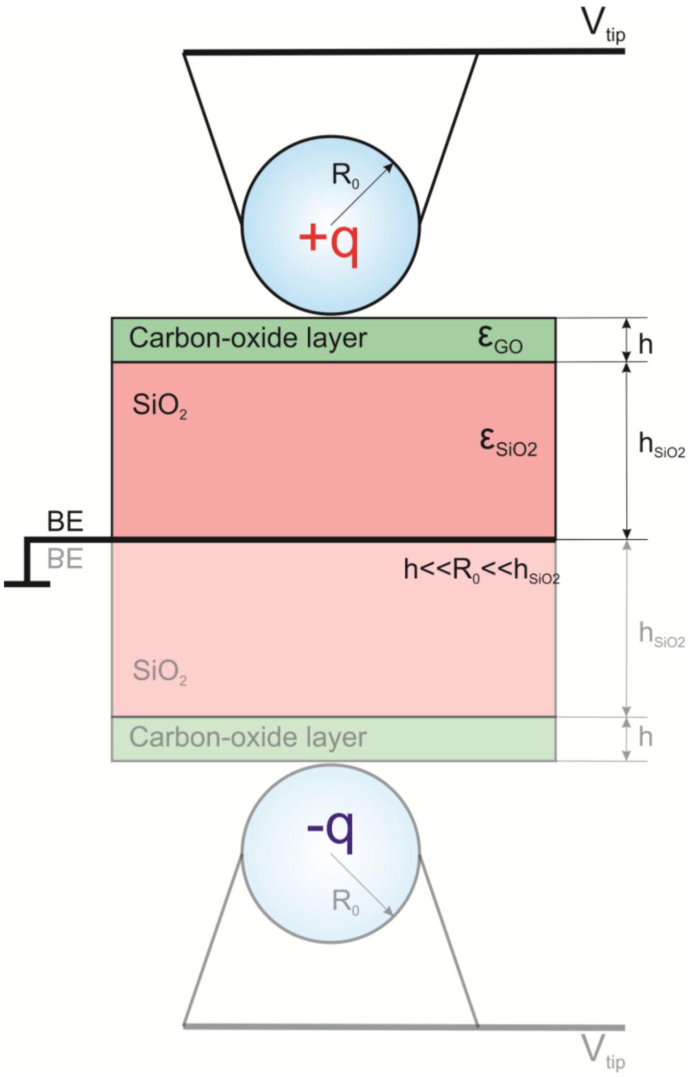

Supplementary Figure 5. Schematic presentation of the tip by the +q charged sphere of the radius R_0, the carbon-oxide dipole layer, the SiO_2 layer, and the mirror charge -q below the back electrode (BE). $h = 0.3\,\text{nm}$, $R_0 = 30\,\text{nm}$, $h_{SiO_2} = 1400\,\text{nm}$.

Supplementary Note 1 Raman spectroscopy measurements

Typical Raman spectrum of single-layer graphene

The typical Raman spectrum of the single-layer graphene (SLG) consists of 3 main lines: D, G and 2D bands[1] (Supplementary Figure 1). The D-band (at about 1350 cm^{-1}) is due to the breathing vibrations of sp^2 carbon rings and requires defects for its activation in the spectrum. The doubly degenerated G-band (at about 1580 cm^{-1}) corresponds to the in-plane vibrations of sp^2 carbon atoms and is ideal to study in-plane stresses and strains. The 2D-band (at about 2672 cm^{-1}) is the second order of the D-band. This peak, being a single in SLG, splits into several peaks in bilayer graphene.

Estimation of graphene quality

High confocality of the microscope makes the Raman lines intensities dependent on the surface relief. Therefore, for accurate analysis of the G and 2D-band variations of graphene, all the spectra were normalized by the maximum value of the Si first order Raman line (Supplementary Figure 1). This eliminates the contribution of the relief and allows comparing the spectra. Integrated intensity and FWHM of the G and 2D-bands were determined by the Lorentz curve fitting of the spectral lines at each point of the studied area, and corresponding Raman maps were constructed (Supplementary Figure 2 and Supplementary Figure 3).

Raman maps in Supplementary Figure 2(a) and Supplementary Figure 2(b) demonstrate periodic variations of the integrated intensity of both G and 2D-bands. The period of these variations coincides with the period of the Si grating, whereas lines' intensity increases on suspended graphene and decreases on supported one. Decrease of the intensities of both G and 2D-bands on the supported graphene can be attributed to interaction between the graphene and the substrate. This assumption is confirmed by the slight increase of FWHM of the G-band (Supplementary Figure 3(a)) on the supported graphene, which demonstrates an increasing disorder in the graphene as a result of its interaction with the SiO_2.

The D- to G-band intensities ratio (I(D)/I(G)) is conveniently used as a footprint of amount of defects in graphene.[1,2] Therefore, bright features in Supplementary Figure 2(c) can be attributed to domain boundaries separating areas with different orientation of carbon lattice – the most common one-dimensional defect in the CVD grown graphene.[2,3]

Spectrum of completely black regions visible on all Raman maps (better seen in Supplementary Figure 3(a)) doesn't contain G and 2D-bands at all, thus pointing out the holes in the graphene sheet. Oblong spots around the holes with large intensity of the G-band can be attributed to graphene wrapped edges, whereas bright linear features in I(G) map – to wrinkles on the graphene surface appeared during the sheet transfer from the copper substrate onto the Si/SiO_2 grating.

In contrast to the G-band, FWHM of the 2D-band is mainly constant all over the studied area (Supplementary Figure 3(b)). The FWHM(2D) increases both around the holes in the graphene and in the small spot in the middle of the map. Fragments of the spectra outside (top spectrum) and inside (bottom spectrum) the spot are presented in Supplementary Figure 3(c). Shape of the 2D-band outside the spot represents a single peak, whereas inside the spot it splits into several peaks, which is characteristic for bilayer graphene.[1] Thus, the largest part of the surface is covered by the single layer graphene.

Estimation of mechanical stresses on the graphene by Raman spectroscopy

Previous works show that the G-band in SLG shifts with applied uniaxial stress.[1,3-5] The rate of change of the G-band position with the applied strain $\frac{\partial \omega_G}{\partial \varepsilon}$ is linear[4]:

$$\frac{\partial \omega_G}{\partial \varepsilon} = k \,. \tag{1}$$

Integration of this expression allows calculating the shift of the G-band position under the external stress:

$$\Delta\omega = \omega - \omega_0 = k\varepsilon \,. \tag{2}$$

Strain dependence of the G-band position depends on a way of graphene creation. Tensile strain in exfoliated graphene leads to $\frac{\partial \omega_G}{\partial \varepsilon} = -49.3$ cm^{-1}, whereas the same strain in CVD graphene leads to $\frac{\partial \omega_G}{\partial \varepsilon} = +41.1$ cm^{-1}.[3] Such essential difference was attributed by authors to additional rotation and slippage of domains with different orientation of carbon lattice, which are absent in exfoliated graphene.[3] Worth noting that the value of $\frac{\partial \omega_G}{\partial \varepsilon}$ also depends on various other experimental conditions such as laser wavelength, polarization direction, etc. Therefore, all quantitative results described below should be considered as estimates.

Combining Supplementary Equations 1 and 2, the value of strain in SLG can be estimated by measuring change of the G-band position in strained ω_s and unstrained ω_0 states:

$$\varepsilon = \frac{(\omega_s - \omega_0)}{\partial \omega_G / \partial \varepsilon} \,. \tag{3}$$

Estimation of the mechanical strains by Supplementary Equation 3 requires an initial (unstrained) position ω_0 of the G-band. Influence of the substrate material on positions of the G and 2D-bands in Raman spectra of mechanically cleaved SLG was investigated in Supplementary Reference 6. For this kind of graphene deposited on Si (as well as on SiO_2) substrate the position of the G-band was defined as 1580 cm^{-1}, but for CVD graphene this value

can be different. Therefore, Raman spectra were measured in 10 points out of the grating region, where the strain is assumed to be zero. Fitting of these spectra allowed us to obtain the average position of the G-band ω_0 equal to 1585.6 cm^{-1} (dashed line in Supplementary Figure 4(b)) and estimate the value of the strains (right axis in Supplementary Figure 4(b)). Since the lowest position of the G-band nowhere reaches the initial value, we concluded that graphene sheet is stressed in greater or lesser degree at each point of the grating.

Spatial distribution of the strains (strain map) is shown in Supplementary Figure 4(a). The strain measurements in Supplementary Figure 4 originally correspond to an average value of both vector components, perpendicular and parallel to the grating structure directions. However, alternating of the strain values in graphene as shown in Supplementary Figure 4(b) can be attributed to the component perpendicular to the grating structure. If the parallel to the grating structure strain component is present, it should induce lines of high stresses (distortions) and, as a result, induce ruptures in the SLG elongated parallel to the grating structure. Since no ruptures and no lines of high stresses along the grating structure were observed (Supplementary Figure 4) we conclude that the strain change in Supplementary Figure 4(b) mainly corresponds to the perpendicular to the grating structure strain component. Strain over the big area with graphene defects varies in a wide range from -7.8 % up to +7.8 %. Positive sign here corresponds to tension of the graphene, whereas the negative one corresponds to compression. The compressive stresses are mainly localized around holes in graphene and along the lines imputed to wrinkles.

Tensile strains occur in defect-free regions of the graphene. The largest values of such strains fall on the supported graphene (shaded rectangles in Supplementary Figure 4(b)) and are about 2.5 times greater than for the suspended regions (about 2%). Strains at the grating ridges are distributed more or less uniformly and demonstrate sharp decreasing on the valleys.

Variations of the G-band position were investigated along the blue line marked in Supplementary Figure 4(a). This line falls into the region with low defect concentration and reflects the real band shift due to the grating effect. For more accurate analysis the spectra were averaged over the adjacent points at the distance of 0.25 μm above and below the line. We found that the G-band position in the supported regions is up to 1.4 cm^{-1} larger, than in the suspended (Supplementary Figure 4(b)). This effect can be attributed to strains appeared in the grating modulated graphene sheet.

Supplementary Note 2 Electrostatic equation for tip potential

To calculate the piezoelectric coefficient, we need to know electric field E in the carbon oxide layer (Supplementary Figure 5). Consider electric field below the tip in the thin carbon-oxide dipole layer with $h << R_0$, where R_0 is the tip radius. Poisson's equation in uniform dielectric medium with the dielectric constant ε is: $\Delta\varphi = -\frac{\rho}{\varepsilon\varepsilon_0}$, where $\varepsilon_0 = 8.854187817 \times 10^{-12}$ F/m is the vacuum permittivity. Corresponding electric field is $E = \nabla\varphi$. The potential generated by a general charge-distribution $\rho(r)$ is:

$$\varphi(r) = \iiint_V \frac{\rho(r')}{\varepsilon\varepsilon_0 |\bar{r} - \bar{r}'|} dr' . \tag{4}$$

For N particles of charge q_i located at points $\bar{r}_i$:

$$\varphi(r) = \sum_{i=1}^{N} \frac{q_i}{\varepsilon\varepsilon_0 |\bar{r} - \bar{r}_i|} . \tag{5}$$

In the model where tip is considered as a sphere of radius R_0 we can use a relation of potential for one particle with the charge located at the center of the sphere (Supplementary Figure 5). Full applied voltage ($V_{tip} = 1$V) corresponds to the voltage drop on the distance ($h + h_{SiO_2}$) from the tip's sphere to the back electrode (BE). Then the electrostatic equation for the tip potential is:

$$V_{tip} = \frac{q_{tip}}{\varepsilon_0} \left(\begin{array}{l} \frac{1}{\varepsilon_{GO} R_0} - \frac{1}{\varepsilon_{GO}(R_0 + h)} + \frac{1}{\varepsilon_{SiO_2}(R_0 + h)} - \frac{1}{\varepsilon_{SiO_2}(R_0 + h + h_{SiO_2})} + \\ + \left(\begin{array}{l} \frac{1}{\varepsilon_{SiO_2}(R_0 + h + h_{SiO_2})} - \frac{1}{\varepsilon_{SiO_2}(R_0 + h + 2h_{SiO_2})} + \\ + \frac{1}{\varepsilon_{GO}(R_0 + h + 2h_{SiO_2})} - \frac{1}{\varepsilon_{GO}(R_0 + 2h + 2h_{SiO_2})} \end{array} \right) \end{array} \right) , \tag{6}$$

where ε_{SiO_2} and ε_{GO} are dielectric constants for the SiO_2 and the carbon-oxide dipole layer, correspondingly. Thickness, h_{SiO_2}, of the SiO_2 layer is 1400 nm, which is much higher than $R_0 = 30$ nm, therefore we can neglect the term $\frac{1}{\varepsilon_{SiO_2}(R_0 + h + h_{SiO_2})} \to 0$ and the last four terms in the big brackets, $\left(\begin{array}{l} \frac{1}{\varepsilon_{SiO_2}(R_0 + h + h_{SiO_2})} - \frac{1}{\varepsilon_{SiO_2}(R_0 + h + 2h_{SiO_2})} + \\ + \frac{1}{\varepsilon_{GO}(R_0 + h + 2h_{SiO_2})} - \frac{1}{\varepsilon_{GO}(R_0 + 2h + 2h_{SiO_2})} \end{array} \right) \to 0$, describing contribution from the mirror charge of the back electrode (Supplementary Figure 5). Then Supplementary Equation 6 takes the form:

$$V_{tip} \approx \frac{q_{tip}}{\varepsilon_0}\left(\frac{1}{\varepsilon_{GO}R_0} - \frac{1}{\varepsilon_{GO}(R_0+h)} + \frac{1}{\varepsilon_{SiO_2}(R_0+h)}\right) \tag{7}$$

The electric field below the tip in the layer is:

$$E_r = \frac{\partial\varphi}{\partial r} = \frac{q_{tip}}{\varepsilon_0}\frac{1}{\varepsilon_{GO}r^2}, \tag{8}$$

$\frac{q_{tip}}{\varepsilon_0}$ can be found from Supplementary Equation 7 as follows:

$$\frac{q_{tip}}{\varepsilon_0} = \frac{V_{tip}}{\frac{1}{\varepsilon_{GO}R_0} - \frac{1}{\varepsilon_{GO}(R_0+h)} + \frac{1}{\varepsilon_{SiO_2}(R_0+h)}} = V_{tip}\frac{\varepsilon_{GO}\varepsilon_{SiO_2}R_0(R_0+h)}{h\varepsilon_{SiO_2} + R_0\varepsilon_{GO}}.$$

Substituting it into Supplementary Equation 8 gives:

$$E = V_{tip}\frac{\varepsilon_{GO}\varepsilon_{SiO_2}R_0(R_0+h)}{h\varepsilon_{SiO_2} + R_0\varepsilon_{GO}}\frac{1}{\varepsilon_{GO}r^2} = V_{tip}\frac{\varepsilon_{SiO_2}(R_0+h)R_0}{(h\varepsilon_{SiO_2} + R_0\varepsilon_{GO})r^2}.$$

The average field within the GO layer below the tip is integral over the GO layer thickness *h* divided by *h*:

$$\begin{aligned}
\frac{1}{h}\int_{R_0}^{R_0+h} E\,dr &= \frac{1}{h}\frac{V_{tip}\varepsilon_{SiO_2}(R_0+h)R_0}{(h\varepsilon_{SiO_2} + R_0\varepsilon_{GO})}\int_{R_0}^{R_0+h}\frac{1}{r^2}dr = \\
&= \frac{1}{h}\frac{V_{tip}\varepsilon_{SiO_2}(R_0+h)R_0}{(h\varepsilon_{SiO_2} + R_0\varepsilon_{GO})}\left(\frac{1}{R_0} - \frac{1}{R_0+h}\right) = \frac{1}{h}\frac{V_{tip}\varepsilon_{SiO_2}(R_0+h)R_0}{(h\varepsilon_{SiO_2} + R_0\varepsilon_{GO})}\frac{h}{R_0(R_0+h)} = . \\
&= \frac{V_{tip}\varepsilon_{SiO_2}}{h\varepsilon_{SiO_2} + R_0\varepsilon_{GO}} = \frac{V_{tip}\varepsilon_{SiO_2}}{R_0\varepsilon_{GO}}\left(1 + \frac{h\varepsilon_{SiO_2}}{R_0\varepsilon_{GO}}\right)^{-1}
\end{aligned} \tag{9}$$

Typical distance between graphene layers in graphite is 2.5 – 3 Å. Assuming $h \approx 0.3\,\text{nm}$ and $R_0 = 30$ nm, $\frac{h\varepsilon_{SiO_2}}{R_0\varepsilon_{GO}} \approx 0.01 << 1$, $\left(1 + \frac{h\varepsilon_{SiO_2}}{R_0\varepsilon_{GO}}\right)^{-1} \approx 1 - \frac{h\varepsilon_{SiO_2}}{R_0\varepsilon_{GO}}$ and Supplementary Equation 9 takes the form:

$$\frac{1}{h}\int_0^h E\,dh' \approx \frac{V_{tip}\varepsilon_{SiO_2}}{R_0\varepsilon_{GO}}\left(1 - \frac{h\varepsilon_{SiO_2}}{R_0\varepsilon_{GO}}\right) \approx \frac{V_{tip}\varepsilon_{SiO_2}}{R_0\varepsilon_{GO}}.$$

This form is valid with accuracy of about $\frac{h\varepsilon_{SiO_2}}{R_0\varepsilon_{GO}} \approx 0.01$, i.e. 1%, and the average electric field below the tip can be expressed as:

$$E = \frac{\varepsilon_{SiO_2}}{\varepsilon_{GO}}\frac{V_{tip}}{R_0} \tag{10}$$

SUPPLEMENTARY REFERENCES

1. Ferrari, A. C. & Basko, D. M. Raman spectroscopy as a versatile tool for studying the properties of graphene. *Nat. Nanotechnol.* **8**, 235–46 (2013).

2. Song, H. S. et al. Origin of the relatively low transport mobility of graphene grown through chemical vapor deposition. *Sci. Rep.* **2**, 337 (2012).

3. Bissett, M. A., Izumida, W., Saito, R. & Ago, H. Effect of domain boundaries on the Raman spectra of mechanically strained graphene. *ACS Nano* **6**, 10229–10238 (2012).

4. Frank, O. et al. Development of a universal stress sensor for graphene and carbon fibers. *Nat. Commun.* **2**, 255 (2011).

5. Ni, Z. H. et al. Uniaxial strain on graphene: Raman spectroscopy study and band-gap opening. *ACS Nano* **2**, 2301–2305 (2008).

6. Wang, Y. et al. Raman studies of monolayer graphene: The substrate effect. *J. Phys. Chem. C* **112**, 10637–10640 (2008).
